# Luminescence Quenching Behavior of Hydrothermally Grown YVO_4_:Eu^3+^ Nanophosphor Excited under Low Temperature and Vacuum Ultra Violet Discharge

**DOI:** 10.3390/ma13153270

**Published:** 2020-07-23

**Authors:** Mihye Wu, Hyemin Park, Eun Gyu Lee, Sanghun Lee, Yu Jin Hong, Sungho Choi

**Affiliations:** 1 Energy Materials Research Center, Korea Research Institute of Chemical Technology, 141 Gajeongro, Yuseong, Daejeon 34114, Korea; wumihye@krict.re.kr (M.W.); ekrmek95@krict.re.kr (H.P.); emmett28@krict.re.kr (E.G.L.); sanghun@krict.re.kr (S.L.); letv97@krict.re.kr (Y.J.H.); 2 Department of Chemical and Biomolecular Engineering (BK-21 Plus), Korea Advanced Institute of Science and Technology (KAIST), Daejeon 34141, Korea; 3 Department of Materials Science and Engineering, Korea University, Seoul 136-701, Korea

**Keywords:** nanophosphor, yttrium vanadate, low temperature photoluminescence

## Abstract

The luminescence quenching behavior and energy transfer process in hydrothermally grown Eu^3+^-doped YVO_4_ nanophosphors were studied using low temperature photoluminescence spectroscopy. The luminescence efficiency of nanophosphor is dependent on the acidity of its solution media and the post annealing condition after hydrothermal processing. The overall results suggest that the abnormal luminescence behavior of Eu^3+^-doped nanocrystalline YVO_4_ under low temperature photoexcitation is due to the incorporated non-radiative hydroxyl groups often encountered in hydrothermal synthesis as well as to the inefficient energy transfer to luminescent ions from vanadate groups.

## 1. Introduction

Yttrium orthovanadate, YVO_4_, is extensively used in laser hosts, lighting devices and displays due to its good thermal stability and advantageous mechanical and physical properties. In addition, it is well-known as one of the phosphor-producing host compounds incorporating several lanthanide ions [1,2,3]. Among these, Eu^3+^-activated YVO_4_ is widely used as a red-emitting phosphor in cathode ray tubes (CRTs) and as an image scanning scintillator [4,5,6,7].

As for the phosphors, rare earth vanadates, especially bulk Eu^3+^-activated YVO_4_, have been shown to be highly efficient photoluminescent materials, with a 70% luminescence quantum yield of the f–f transition, giving them the best luminescence efficiency among all candidate materials when excited by ultraviolet (UV) sources [8,9,10,11,12]. Additionally, the research on nanoscale synthesis phosphates and vanadates followed by the applications to luminescent centers was widely performed [13]. These ortho-phosphates and vanadates have lower formation energy than other oxide compounds, thus allowing their synthesis as nanoparticles through colloidal chemistry while preventing agglomeration of nanoparticles at higher reaction temperatures [14,15].

The corresponding lanthanide-ion activated nanophosphors, however, are still underway owing to their lower quantum efficiency and long term stability compared to those of micron scale phosphors. It is normally induced by the high surface/volume ratio with increasing non-radiative surface trapped charge densities, which finally leads to luminescence quenching. Therefore, many efforts aiming at the application of these materials concentrate on the deactivation of surface quenching, which can be achieved by surface modifications for the given nanophosphors like as the coordinated ligands and/or by the encapsulation with passivating shells [13]. Notably, as far as could be determined, there have not been any previous reports of the low temperature and vacuum UV (VUV) radiation excited luminescence process of Eu^3+^-activated YVO_4_ nanophosphors in conjunction with the surface trap centers.

In this contribution, the YVO_4_:Eu^3+^ nanophosphors are synthesized and their luminescence quenching behavior excited under the harsh conditions, like as very low temperature (descending down to five Kelvin) and vacuum radiation (λ_ex_ = 147 nm) is characterized. We have reported a polyethylene glycol (PEG)-passivated luminescence enhancement of the corresponding YVO_4_:Eu^3+^ nanophosphor as well as the controlled synthesis of nanophosphor [16,17]. Compared to the previous works, in the present work, the temperature effects on the luminescent characteristics of Eu^3+^ in YVO_4_ host and correlation between energy transfer from the host to the activators center and to radiation traps in nanocrystalline YVO_4_:Eu^3+^ phosphor are evaluated. In addition, the given phosphor layer is excited under the VUV plasma gas discharging, which can visualize the luminescence quenching behavior for the surface-mediated nanophosphors.

## 2. Materials and Methods

High-purity Y_2_O_3_ (99.99%, Sigma-Aldrich, St. Louis, MO, USA), V_2_O_5_ (99.99%, Junsei Chem., Tokyo, Japan) and Eu_2_O_3_ (99.99%, Sigma-Aldrich) were chosen as the starting materials. First, 0.4547 g V_2_O_5_ was dissolved in diluted nitric acid, and then stoichiometric amounts of Y_2_O_3_ (0.5364 g) and Eu_2_O_3_ (0.044 g) were added to the corresponding solution. Ammonia solution (NH_4_OH, 28% in H_2_O, Sigma-Aldrich) was added dropwise until the pH of the two different solutions reached 7 and 10, respectively. After continuous stirring for 30 min, 100 mL of the mixture was transferred into an autoclave vessel. The hydrothermal reaction was undergone at 200 °C for 24 h, and then, the furnace was cooled to room temperature. The as-synthesized powders were separated by centrifugation followed by washing with ethanol and drying at 80 °C for 10 h.

The phase composition of these phosphors was determined using an XRD-Rigaku DMAX-33 X-ray diffractometer (Tokyo, Japan) with Cu Kα radiation. The operation voltage and current were 40 kV and 40 mA, respectively. The morphology of the obtained samples was studied with a FEI-TECNAI G^2^ transmission electron microscope (TEM, Hillsboro, OR, USA) operated at 200 kV. The Fourier transform infrared spectra (FT-IR, Nicolet 6700, ThermoScientific, Waltham, MA, USA) were recorded using pellets with KBr. The UV emission spectra were measured using a luminescence spectrometer (Luminescence spectrometer, PSI trading, Suwon, Korea) at room temperature with a xenon lamp as an excitation source. The molar concentration of Eu^3+^ was adjusted to 5% in YVO_4_ that is the optimal concentration for producing the maximum possible fluorescence efficiency for Eu^3+^ in this host. In addition, the temperature quenching was measured while continuously varying the temperature from room temperature to about 5 K under the excitation by a He–Cd laser (325 nm, IK series, Kimmon Koha, Tokyo, Japan).

Finally, using plasma discharge driven test panels, we examined the VUV-excited luminescence behavior of the nanophosphors. The experimental conditions were the same as those in our previous work [16,18]. The test panel was composed of two glass plates; the front one on which the electrodes and dielectric layers are coated and the other one with the nanophosphor-coated. Those two plates were piled up with the gap of 2 mm followed by filling with a Ne-20% Xe mixture gas. Then, the pulsed AC power with a frequency of 30 kHz and a voltage of 290 V was applied to initiate the glow discharge conditions.

## 3. Results

**Figure 1:**
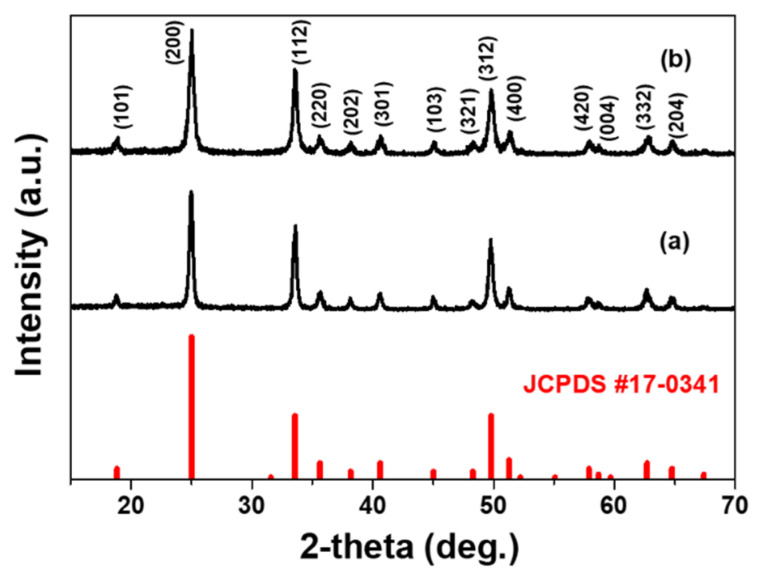
XRD patterns of the hydrothermally grown YVO_4_:Eu^3+^ nanoparticles using the precursor solutions at (a) pH = 7 and (b) pH = 10.

The degree of crystallinity and the crystal structure of hydrothermally grown nanophosphors were analyzed. Compared to the reference data (JCPDS 17-0341), as shown in Figure 1, each compound has the typical diffraction peaks of the tetragonal with *I*_1_/*amd*_1_ space group YVO_4_ compound. Both the samples have identical crystalline structure, and, thus, the precursor solution acidity (amount of ammonium solution) has a little influence on the phase formation of the corresponding phosphor host materials. The additional XRD results for nanoparticles as-synthesized at different pH values clearly show the YVO_4_ structure (JCPDS 17-0341) (Figure S1). The crystal size was estimated using the Scherrer equation, and the average values for the as-synthesized particles were around 30–50 nm.

**Figure 2:**
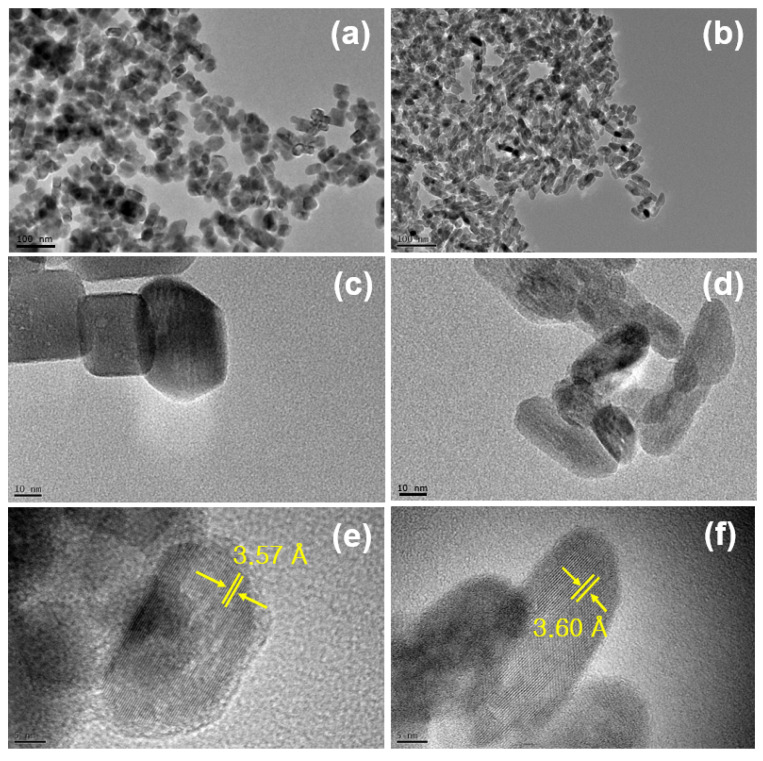
Corresponding TEM images of the YVO_4_:Eu^3+^ nanoparticles grown using the precursor solutions with (**a**,**c**,**e**) for pH = 7 and (**b**,**d**,**f**) for pH = 10.

The size, morphology and crystallinity of the as-prepared nanophosphor were evaluated by TEM, and the results are shown in Figure 2. Please note that the some TEM images (specially pH = 7) are quoted from our previous work [16]. It can be seen that the hydrothermally grown YVO_4_:Eu^3+^ crystals had polygonal shapes with their mean diameter was ca. 25 nm, which is similar to values estimated from the XRD pattern using the Scherrer formula. Obviously, the crystallinity was somewhat different with precursor solution acidity, which controls the overall luminescence efficiency under photoexcitation. Generally, the nanoparticle nucleation and growth kinetics are influenced by the pH values in solution chemistry. The luminescence property of the given YVO_4_:Eu^3+^ are substantially dependent on the hydrothermal synthesis conditions. The supporting data shown in Figures S2 and S3 clearly display that the uniform particle formation with characteristic Eu^3+^-activated red emission is proceeded in more basic solutions, pH > 7 In our previous work, the PEG-assisted hydrothermal YVO_4_:Eu^3+^ nanophosphor was obtained with different morphology and modified surface structure, assisting the formation of specific morphology with stable surface conditions followed by an enhanced luminescence property [17]. In this work, however, we do focus on the comparative study between the two samples, formed at pH = 7 and 10, since the surface states are distinguishable, and, thus, we can evaluate the surface-related luminescence quenching behavior.

**Figure 3:**
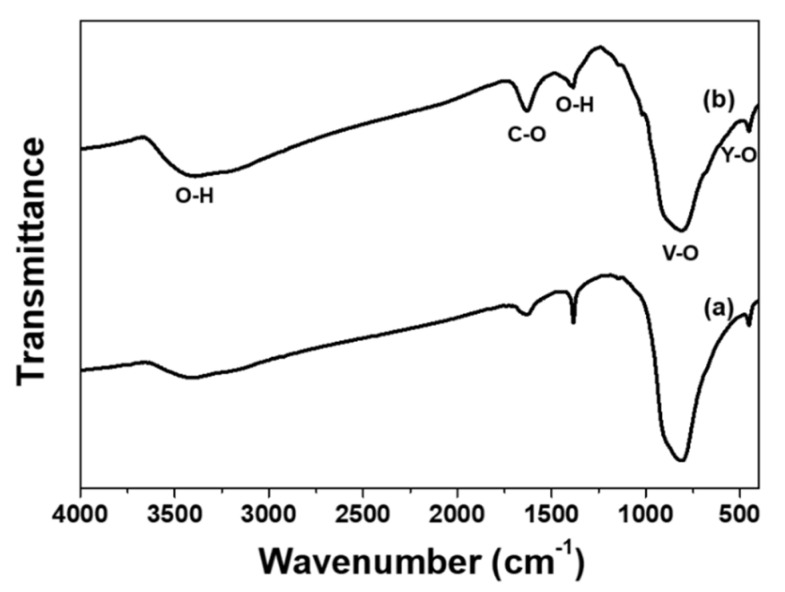
FT-IR spectra of YVO_4_:Eu^3+^ nanoparticles hydrothermally grown using the precursor solutions at (a) pH = 7 and (b) pH = 10.

The chemical information and surface residual species were investigated by FT-IR spectroscopy, as shown in Figure 3. The spectra are recorded from the samples as-synthesized with different solution acidity pH = 7 (a) and pH = 10 (b). The strong peak around 815 cm^−1^ and peak at 450 cm^−1^ are apparently associated with the YVO_4_ host compound, which stem from the vibrational modes of the V–O and the Y–O bonds, respectively [19]. The presence and strong peaks of these characteristic modes found for the given hydrothermally grown nanoparticles indicate that optically stable YVO_4_ host is successfully obtained, which is in good agreement with the XRD results. The absorption bands of residual H–O–H (ca. 1630 cm^−1^) and hydroxyl groups (ca. 3400 cm^−1^) are clearly observed in both samples, these species are generally known as the luminescent quenching centers. It can be seen that post-annealing at high temperatures (not shown here) can substantially eliminate the surface adsorbed hydroxyl components as well as NH4^+^ band positioned at 1380 cm^−1^, which finally leads to an enhanced luminous efficacy in nanocrystalline phosphors. The hydroxyl ions are assumed to enter the nanocrystals from the aqueous media during the hydrothermal process and the concentration of hydroxyl ions is relatively high when the samples were prepared using solutions with a higher pH value, as shown in Figure 3b. The correlation between the emission efficiency and surface adhesive NH_4_^+^ groups in still ambiguous. Nevertheless, such a controllable and deactivated surface treatment will significantly stabilize the luminescent properties of the nanoparticles under photo-excited conditions. This is discussed in more detail using low temperature luminescence results.

**Figure 4:**
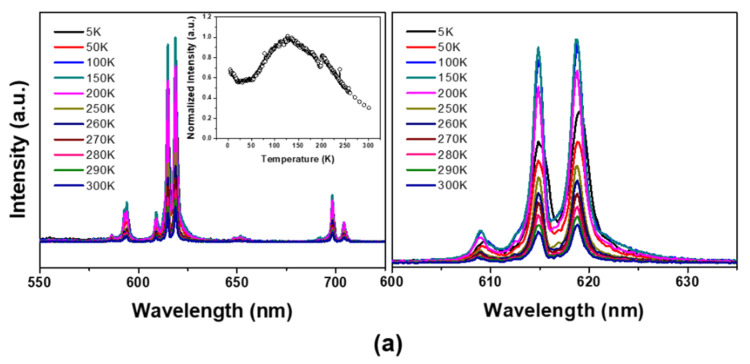

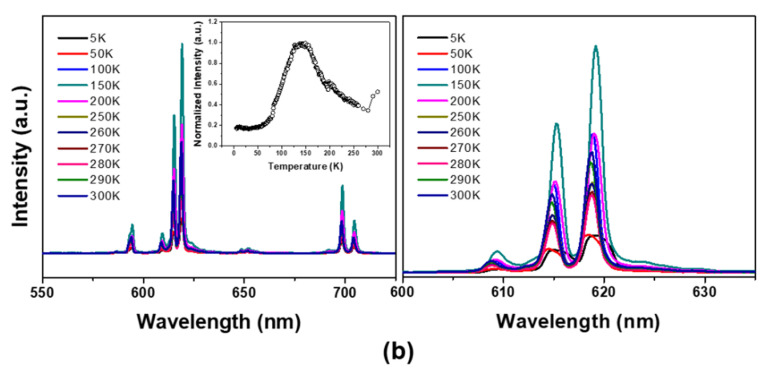
Temperature dependent PL spectra of YVO_4_:Eu^3+^ nanophosphors hydrothermally grown using the precursor solutions at (**a**) pH = 7 and (**b**) pH = 10. Corresponding intensity change of ^5^D_0_→^7^F_2_ transition (inset image) and enlarged emission spectra are also presented.

The temperature-dependent emission spectra of the corresponding nanophosphors are presented in Figure 4. First, upon the He–Cd laser excitation at room temperature (T = 300 K), the Eu^3+^-doped YVO_4_ nanoparticles give a strong red-emission from the ^5^D_0_–^7^F_J_ transition of Eu^3+^ [10,11,12]. The Eu^3+^-doped YVO_4_ nanoparticles also exhibit a broad excitation band positioned at 312 nm, which is not shown, corresponding to charge transfer from oxygen to the central vanadium atom inside ${\mathrm{VO}}_{4}^{3-}$anions within overall temperature ranges. The site symmetry and electric dipole transitions between the f-electron levels of Eu^3+^ in the nanocrystalline YVO_4_ host are similar to those of micron scale phosphor, which induce the excitation and efficient energy transfer to the Eu^3+^ ions from charge-transfer states involving the V–O component of the lattice.

The emission spectra and intensity change of the corresponding emission peak positioned at 619 nm of ^5^D_0_–^7^F_2_ transition are summarized in Figure 4a,b, respectively. The emission behaviors were consistent in each other; that is, emission intensity is maximal at specific temperature (about 125–150 K). Additionally, below 50 K, the emission spectra were somewhat shifted to a longer wavelengths with broadened FWHM. The emission intensity decrease with lowering temperature, called as a low-temperature luminescence quenching, may be related to the thermally stimulated luminescence (TSL) phenomena of the nanophosphor and/or defect-inherited luminescent materials [20,21,22]. The abrupt decreased emission intensity for the nanophosphor synthesized at pH = 10 with higher surface trap density, as shown in Figure 4b, clearly indicates the surface-induced luminescence quenching. Thus, the luminescence quenching upon cooling was understood by the surface-trapped charge carriers at low temperatures.

In some cases, the luminescence efficiency of the lanthanide-activated phosphors is closely related to the energy transfer efficiency between hosts to activators via those thermally activated luminescence process. T. Arai et al., have reported the low-temperature luminescence behavior of the chemical etching, followed by post-annealed SnO_2_:Eu^3+^ phosphor, in which the overall thermal quenching can be understood only by the energy transfer between the host lattice and activators [23]. On the contrary, in our results, the substantially decreased luminescence intensity of the as-synthesized YVO_4_:Eu^3+^ nanophosphor (i.e., activating the surface-trap sites) above 150 K may be attributed to a competing transition probability between the activator and the surface recombination centers such as an hydroxyl group. As is well-known, the electric dipole transition is spatially allowed only to meet the local symmetry site conditions of the lanthanide ions. Thus, abnormal emission behavior indicates that more Eu^3+^ ions positioned at non-inversion symmetry sites in nanocrystalline YVO_4_ compared to the microscale phosphors, which prevails at ranges from 5 to 150 K.

Furthermore, the substantially decreased luminescence efficiency of the as-synthesized sample above 150 K is attributed to a competing transition probability between the activator and the surface recombination center. This appears to match the findings of Gapontsev et al., who observed decreased emission efficiency under electron-beam excitation over 150 K due to the thermal contact that arises between ^5^D states and charge-transfer states [24]. In Mn^2+^-doped ZnS nanophosphor, Bhargava et al. suggested that the faster energy transfer to the luminescent center in smaller particles than the transfer rate for direct band transition or surface recombination leads to an increasing luminescence efficiency with decrease of particle size [25]. Although the exact luminescence process in nanocrystalline YVO_4_:Eu^3+^ is still unclear, the radiative transition probability of the luminescent center is dominant below 150 K due to the thermally quenched and deactivated non-radiative sites in the case of samples prepared using solutions with higher pH value—which contain more residual surface traps like as hydroxyl (and/or NH^4+^) groups which acts as an non-radiative sites. Thus, the energy transfer from the vanadate host to activator center is more efficient and represents the prevailing overall luminescence process over the low temperature regions. Qualitatively, however, the quenching of emission attributed to thermally promoted transition from ^5^D states to non-radiative sites prevails above this temperature range.

**Figure 5:**
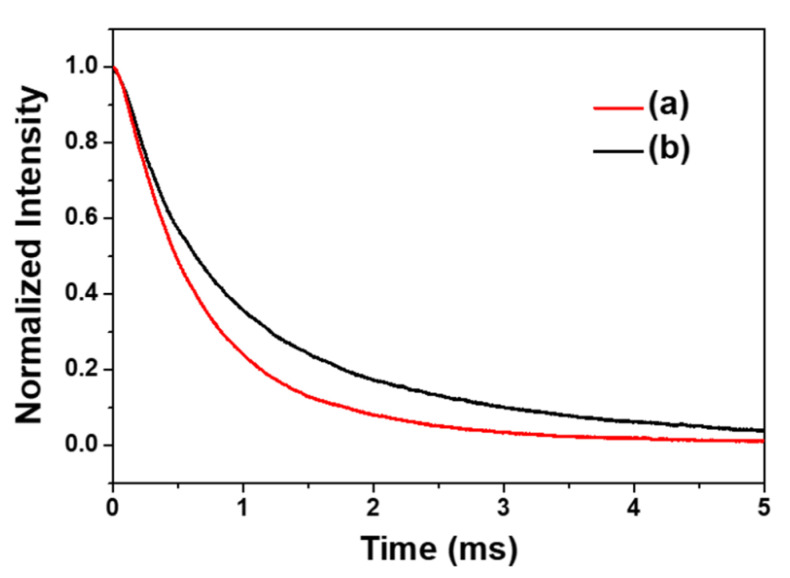
Vacuum UV (VUV) luminescence decay curve of YVO_4_:Eu^3+^ nanophosphors with different solution acidity. (a) pH = 7 and (b) pH = 10.

Generally, the formulation of impurities/defects within the phosphor, serves as carrier trap centers, of which the trapped carriers have the ability to escape from followed by the appearance of delayed-radiation process [26]. As shown in Figure 5, we can realize a longer decay time for nanophosphors with higher trap-density prepared at pH = 10 solution; 2.89 ms for YVO_4_:Eu^3+^ phosphor grown at pH = 10 and 1.76 ms for pH = 7, respectively. Thus, a lower luminescence intensity below 150 K for the sample with higher trap density is well consistent.

**Figure 6:**
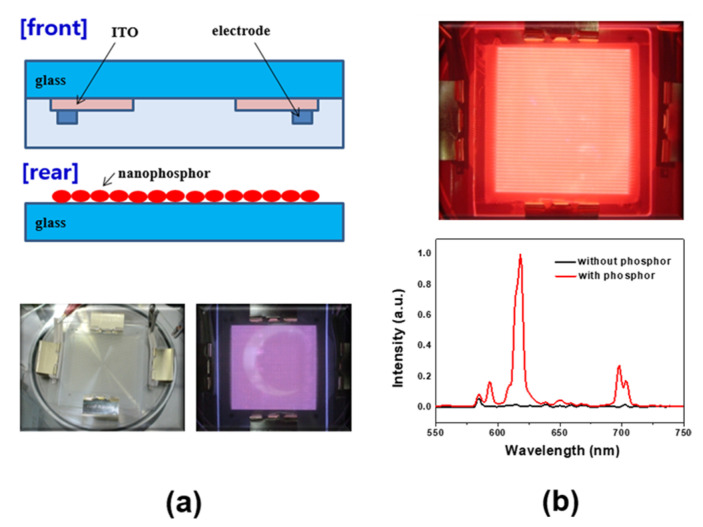
VUV panel test using the given YVO_4_:Eu^3+^ nanophosphors. (**a**) Panel structure and photographs of the bare glass with/without gas glow discharge; (**b**) YVO_4_:Eu^3+^ overcoated layer under Xe/Ne gas discharge and corresponding their emission spectra.

Finally, we fabricated transparent panels for plasma discharge and analyzed the luminance property of the as-prepared nanophosphors. The test panels consisted of front and rear glass plates. A schematic diagram of the test panel using rear plates without a phosphor layer is shown in Figure 6a. Upon plasma discharging, the luminance of the panels coated with YVO_4_:Eu^3+^ nanophosphor increased up ~50 cd/m^2^, while the luminance was below 5 cd/m^2^ for the panel without phosphor layer (Figure 6b) Based on the YVO_4_:Eu^3+^ nanophosphor-illuminating panel with the characteristic red emission, the given nanophosphors are comparable to the micron scale red emitting phosphors even under VUV radiations.

## 4. Conclusions

In this study, the low temperature luminescence properties of hydrothermally grown YVO_4_:Eu^3+^ nanocrystals were thoroughly examined in conjunction with the surface adhesive species.

The study of YVO_4_:Eu^3+^ nanophosphor with low temperature luminescence quenching is useful for a complete understanding of size and surface chemistry effects induced by different preparation conditions It was found that the photoluminescence efficiency, especially at ≤150 K, is closely related to the surface trap centers of the hydrothermally grown nanophosphors like as hydroxyl groups. It can be understood by the increasing non-radiative energy transfer through the coupling of the ^5^D_0_ states of the Eu^3+^ ions to the O–H vibration states. This finding underscores the impact of surface-bounded OH^−^ groups on the quenching of the excited state of Eu^3+^, especially in nanoscale phosphors with temperature-dependent luminescence below 150 K. We can realize that the use of highly luminescent lanthanide ion-activated YVO_4_ nanophosphor is promising one even the surface-mediated luminescence quenching property is overwhelmingly activated.

## Supplementary Materials

The following are available online at https://www.mdpi.com/1996-1944/13/15/3270/s1, Figure S1: XRD patterns for the hydrothermally grown YVO_4_:Eu^3+^ compound with different precursor solution pH, Figure S2: YVO_4_:Eu^3+^ photoluminescence emission intensity change with hydrothermal reaction parameters; precursor pH and reaction time, Figure S3: **T**EM images of the hydrothermally grown YVO_4_:Eu^3+^ compound using the precursor solution with acidic conditions (pH = 7).
