# Supplementary material for: Luminescence Quenching Behavior of Hydrothermally Grown YVO4:Eu3+ Nanophosphor Excited under Low Temperature and Vacuum Ultra Violet Discharge
Source: Materials (Basel). 2020 Jul 23;13(15):3270. doi: 10.3390/ma13153270 (PMC7435471; doi:10.3390/ma13153270)
Supplement: Supplementary file 1 [file materials-13-03270-s001.pdf]

materials

MDPI

Supporting Information

Luminescence Quenching Behavior of Hydrothermally Grown $YVO_4:Eu^{3+}$ Nanophosphor Excited Under Low Temperature and Vacuum Ultra Violet Discharge

Mihye Wu [1,2], Hyemin Park [1], Eun Gyu Lee [1,3], Sanghun Lee [1], Yu Jin Hong [1] and Sungho Choi [1,*]

[1] Energy Materials Research Center, Korea Research Institute of Chemical Technology, 141 Gajeongro, Yuseong, Daejeon 34114, Korea; wumihye@krict.re.kr (M.W.); ekrmek95@krict.re.kr (H.P.); emmett28@krict.re.kr (E.G.L.); sanghun@krict.re.kr (S.L.); letv97@krict.re.kr (Y.J.H.)

[2] Department of Chemical and Biomolecular Engineering (BK-21 Plus), Korea Advanced Institute of Science and Technology (KAIST), Daejeon 34141, Korea

[3] Department of Materials Science and Engineering, Korea University, Seoul 136-701, Korea

* Correspondence: shochoi@krict.re.kr; Tel.: +82-42-860-7372

Received: 14 May 2020; Accepted: 22 July 2020; Published: 23 July 2020

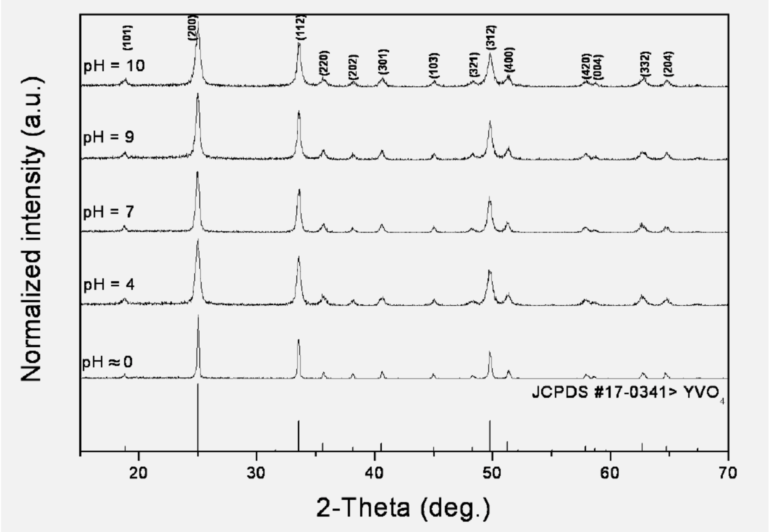

Figure S1. XRD patterns for the hydrothermally grown $YVO_4:Eu^{3+}$ compound with different precursor solution pH levels.

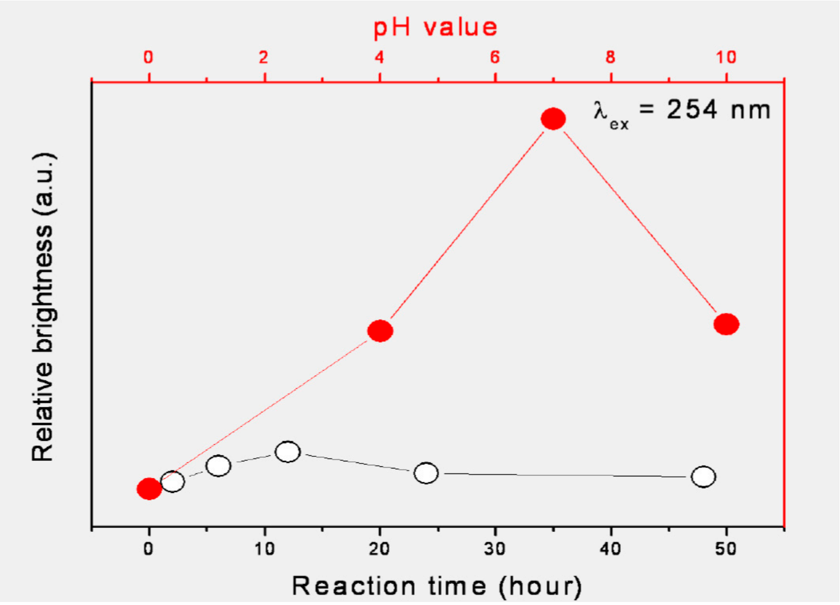

Figure S2. $YVO_4:Eu^{3+}$ photoluminescence emission intensity change with hydrothermal reaction parameters; precursor pH and reaction time.

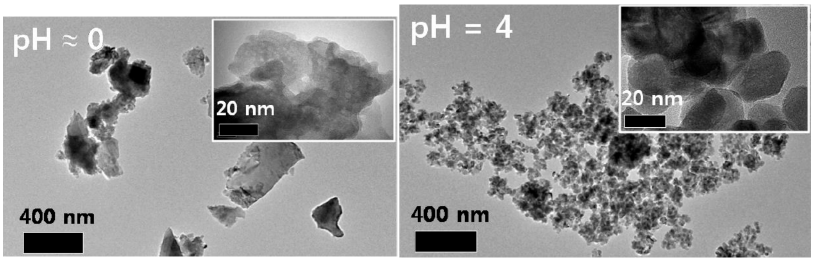

Figure S3. TEM images of the hydrothermally grown $YVO_4:Eu^{3+}$ compound using the precursor solution with acidic conditions (pH = 7).
